# Etude descriptive et analytique du cancer de l’œsophage au Togo

**DOI:** 10.11604/pamj.2014.19.315.4557

**Published:** 2014-11-21

**Authors:** Bouglouga Oumboma, Lawson-Ananissoh Laté Mawuli, Bagny Aklesso, Kaaga Laconi, Redah Datouda

**Affiliations:** 1 Service d’Hépato-Gastroentérologie, Centre Hospitalier et Universitaire de Lomé, Togo

**Keywords:** Cancer, œsophage, épidémiologie, Togo, Cancer, esophagus, epidemiology, Togo

## Abstract

**Introduction:**

Décrire les aspects épidémiologiques, cliniques, endoscopiques et histologiques du cancer de l’œsophage (CO) au Togo.

**Méthodes:**

Il s'agit d'une étude rétrospective descriptive et analytique menée sur 8 ans (Janvier 2005-Décembre 2012) dans le service d'hépato-gastroentérologie (HGE) du CHU Campus de Lomé. Etaient inclus les dossiers des patients hospitalisés pour CO confirmé histologiquement.

**Résultats:**

Sur 8 ans, 24 patients remplissant nos critères d'inclusion ont été retenus soit 3cas de CO par an et 0,55% des hospitalisations. L’âge moyen des patients était de 57,08 ans (extrêmes: 32 et 82 ans). La dysphagie et l’épigastralgie étaient les motifs d'hospitalisation les plus rencontrés. L'alcool (n=15), le tabac (n=13) étaient les facteurs de risque les plus présents. A la fibroscopie, les lésions étaient ulcéro-bourgeonnantes et hémorragiques (n=12), ulcéro-bourgeonnantes (n=5); ces lésions siégeaient au niveau du 1/3 inférieur (n= 11), à l'union 1/3 supérieur 1/3moyen de l’œsophage (n= 13) et aucun au niveau du 1/3 supérieur. Seize lésions étaient des carcinomes épidermoïdes et 3 des adénocarcinomes. L’évolution dans le service a été fatale dans 2cas; 16 patients avaient été transférés en chirurgie pour des soins palliatifs et 5 patients (20,8%) étaient perdus de vue.

**Conclusion:**

Le CO semble en augmentation au Togo. L'alcool et le tabac sont les facteurs de risque et le pronostic sévère dans notre série est lié au retard diagnostic. Son dépistage précoce passe par une consultation rapide devant toute dysphagie chez un sujet de 50 ans et plus.

## Introduction

Le cancer de l’œsophage (CO) est un cancer de diagnostic tardif et à pronostic défavorable en raison de l'absence de dépistage précoce de lésions précancéreuses, liée au retard de consultation [1, 2]. Il est une tumeur maligne primitive dont l'incidence varie selon les pays. Sa fréquence et ses facteurs de risque sont bien connus dans les pays développés [1, 3]. En Afrique plusieurs études ont rapporté des fréquences variables permettant ainsi de classer le CO au 4e rang des cancers chez l'homme après le sarcome de kaposi, le carcinome hépatocellulaire et le cancer de la prostate [3–5]. Au Togo, les fréquences du CO sont disparates laissant ainsi une sous-estimation de la fréquence réelle de cette tumeur maligne primitive. Le présent travail a pour but de décrire les aspects épidémiologiques, diagnostic, évolutifs et histologiques du CO dans le service d'hépato-gastrœntérologie (HGE) du CHU Campus de Lomé.

## Méthodes

Il s'agit d'une étude rétrospective, descriptive et analytique menée sur 8 ans (1er Janvier 2005 au 31 Décembre 2012) dans le service d'HGE du CHU Campus de Lomé. Etaient inclus les dossiers des patients hospitalisés pour CO documenté et confirmé histologiquement. Les données ont été recueillies à partir d'une fiche d'enquête comportant les paramètres épidémiologiques, cliniques, endoscopiques et histologiques. Nos données ont été traitées manuellement.

## Résultats

Au cours de notre période d’étude 4351 patients étaient hospitalisés dans le service dont 24 remplissaient nos critères d'inclusion. Ceci correspond à une fréquence hospitalière de 0,55% des hospitalisations dans le service. L’âge moyen était de 57,08ans (extrême: 32 et 82ans). Sur nos 24 patients, on retrouvait la même proportion d'hommes et de femmes. Le pic de fréquence du cancer de l’œsophage se situait entre 50 et 59 ans pour les deux sexes avec 9 cas dont 5 hommes et 4 femmes. Le motif d'hospitalisation le plus fréquemment retrouvé était la dysphagie dans 58,33% (n=14), suivi des épigastralgies (20, 84%) (Tableau 1). La durée moyenne d’évolution avant l'hospitalisation était de 78,37 jours (extrêmes de 7 et de 365 jours). La durée maximale a été observée chez un sujet de sexe masculin âgé de 58 ans, admis pour dysphagie sur fond d’épigastralgies, évoluant depuis environ un an. L’éthylisme chronique était présent chez dix-neuf patients (79,16%), le tabagisme chronique chez treize (54,17%). L'association alcoolisme et tabagisme chronique était présente chez treize patients (54,17%). La prise quotidienne d'infusions d'herbe a été retrouvée chez 3 patients (12,5%). L’état général était altéré chez 15 patients (62,5%). Une pâleur conjonctivale était retrouvée chez 8 patients (33,33%). Nous avons retrouvé une hépatomégalie douloureuse chez deux patients, et un ganglion de Troisier chez un autre. La fibroscopie œsogastroduodénale(FOGD) réalisée chez tous nos patients, a noté une lésion ulcéro-bourgeonnante et hémorragique chez 12 patients (50%) (Tableau 2). Les lésions siégeaient plus au niveau du tiers moyen de l’œsophage (54,16%) suivi du tiers inférieur (45,84%). Aucune lésion ne siégeait sur le tiers supérieur de l’œsophage (Tableau 3). La biopsie n'avait pu être réalisée chez 5 patients, en raison du risque hémorragique très élevé. Sur le plan histologique le carcinome épidermoïde était retrouvé dans 84,21% des cas (n=16), suivi de l'adénocarcinome dans 15, 79% des cas (n=3). L’échographie abdominale a été faite chez trois patients et retrouvait une hépatomégalie nodulaire chez deux patients. La radiographie du thorax a été faite chez sept patients. Elle était normale dans six cas, et retrouvait des images en faveur de métastases pulmonaires chez un patient. La durée moyenne d'hospitalisation était de 14,75 jours. L’évolution clinique dans le service a été fatale chez 02 patients (8,33%), alors que 16 patients avaient été transférés en chirurgie pour des soins palliatifs et 05 patients (20,8%) étaient sortis contre avis médical.

**Tableau 1:** Répartition des cas de cancer de l’œsophage en fonction du motif d'hospitalisation

Motifs d'hospitalisation	Effectif (n)	Pourcentage(%)
Dysphagie	14	58,3
Epigastralgies	5	20,8
Altération de l’état général	2	8,3
Vomissements	2	8,3
Hémorragie digestive	1	4,1

**Tableau 2:** Répartition des cas de cancer de l’œsophage en fonction de l'aspect macroscopique

Aspects macroscopiques	Effectif (n)	Pourcentage(%)
Ulcéro-bourgeonnant et hémorragique	12	50
Ulcéro-bourgeonnant	5	20,8
Ulcéro-nécrotico-hémorragique	3	12,5
Bourgeonnant et hémorragique	3	12,5
Ulcéro-hémorragique	1	4,2
Total	24	100

**Tableau 3:** Répartition des cas de cancer de l’œsophage selon l'aspect macroscopique en fonction du siège de la lésion

Siège	Ulcéro-bourgeonnant et hémorragique	Ulcéro-nécrotico-hémorragique	Ulcéro-bourgeonnant	Ulcéro-hémorragique	Bourgeonnant et hémorragique	Total
Tiers supérieur	–	–	–	–	–	
Tiers moyen						13
Union 1/3> et1/3X	–	–	1	–	–	
1/3X	8	2	–	–	2	
Tiers inférieur						11
Union 1/3X et 1/3 <	1	1	–	1	–	
1/3 <	3	–	4	–	1	
Total	12	3	5	1	3	24

1/3> : Tiers supérieur 1/3X : Tiers moyen 1/3< : Tiers inférieur

## Discussion

Le cancer de l’œsophage est un cancer de mauvais pronostic et sa gravité est liée au retard de diagnostic. Sa fréquence est diversement appréciée par plusieurs auteurs. Celle-ci est passée de 0,35% à 0,55% des hospitalisations dans notre série avec 24 cas sur 8 ans, soit 3cas par an en moyenne. Des fréquences plus élevée ont été rapportées dans la littérature africaine [5–8]. Cette variabilité des fréquences et la faible incidence dans notre étude pourrait s'expliquer par la mauvaise orientation de nos patients liée au principal motif de consultation de notre série qui était la dysphagie et qui amenait les patients à consulter en oto-rhino-laryngologie (ORL); mais aussi par la pénurie de gastroentérologues au Togo qui n'en compte que 4 pour 6millions d'habitants avec un seul service de référence à Lomé la capitale. L’âge moyen de nos patients était de 57,08 ans, identique aux données de Peghini à Madagascar [9] qui retrouvait un âge moyen de 57,5 ans. Ceci confirme les données de la littérature qui stipulent qu'en Afrique, le cancer de l’œsophage est fréquent surtout à partir de la 5ème décade [3, 5, 9]. Par contre il est supérieur à celui trouvé par Dia et al [10] qui avaient trouvé 49ans. Cliniquement la dysphagie considérée par plusieurs auteurs [3, 5, 10] comme le symptôme d'appel le plus fréquent du cancer de l’œsophage, a été le motif d'hospitalisation le plus fréquent dans notre étude. L'apparition de ce symptôme signe l’état avancé de la maladie. Dans plusieurs séries [3, 5, 10] comme dans la nôtre, ce symptôme est souvent accompagné d’épigastralgie et d'altération de l’état général. Le reflux gastro-œsophagien(RGO) responsable de l’œ;sophagite de Barrett est un facteur de risque indirect du cancer de l’œsophage mais dans notre série aucun cas n'a été noté. Nos patients avaient consulté entre 3 et 8 mois après l'apparition du premier symptôme. Ce même constat était fait par d'autres auteurs africains [3, 6, 7, 10]. Mais en France certains auteurs [11] avaient trouvé un temps moyen de 2,8 mois. Ce retard de consultation dans nos milieux pourrait s'expliquer d'une part par le niveau socio-économique bas de nos patients qui n'arrivent pas à payer ni la consultation ni les bilans complémentaires entraînant leur orientation vers les tradipraticiens.

Dans notre étude, outre l'altération de l’état général, le principal signe physique retrouvé était la présence de ganglions de Troisier chez un patient. Ceci témoigne de l'extension et de l’état avancé de la maladie et s'explique par la longue durée d’évolution de la maladie avant le diagnostic. L’éthylisme chronique était présent chez 79,17% (n=19) de nos patients et le tabagisme était retrouvé chez 13 malades (54,17%). Ces deux facteurs de risque (alcool-tabac) ont été également retrouvés par Bagny et al [12]. De nombreux auteurs [1, 3, 13] sont unanimes sur le rôle de l'alcool et du tabac dans la genèse de ce cancer. Dans notre étude presque tous nos patients (n=19) étaient éthyliques et treize (n=13) patients étaient tabagiques. L'alcool associé au tabac augmenterait plus de 100 fois le risque de développer ce type de cancer [14]. D'autres facteurs de risque alimentaire comme le poisson fumé, les viandes fumées contenant des nitrosamines ont été incriminés dans la genèse de ce cancer en Afrique, comme en Asie [4]. Dans notre étude ces facteurs alimentaires avaient été également retrouvés chez nos patients mais le plus fréquent était la consommation du maïs par tous nos patients comme aliment de base. En effet ce rôle du maïs avait déjà été évoqué et actuellement la consommation élevée du maïs est corrélée au risque de survenue du cancer de l’œsophage en Afrique par le biais de la contamination du maïs par une mycotoxine, la fumonisine [15]. Les tumeurs siégeaient en majorité au niveau du tiers moyen et tiers inférieur dans notre travail comme l'avaient constaté également d'autres auteurs [3, 6, 10]. Tout comme dans la série de Bagny et al. [12], le tiers supérieur n'a été le siège d'aucune lésion cancéreuse. Cela pourrait s'expliquer par le fait que les cancers du 1/3 supérieur de l’œsophage de par leurs manifestations cliniques amèneraient les patients à consulter et se faire prendre en charge en ORL. La biopsie était réalisée chez tous les patients, le carcinome épidermoïde était la variété la plus retrouvée dans 84, 21% des cas, conforme aux données retrouvées par Sawadogo [7] dans 68,75%. La deuxième variété la plus retrouvée était l'adénocarcinome. Nous n'avons pas retrouvé dans notre série de cas d’épithélioma ou de sarcome. L'alcool et le tabac seraient fréquemment responsables [14] de cette forme histologique alors que le RGO était le facteur de risque indirect le plus fréquent qui était retrouvé chez tous les patients qui avaient présenté un adénocarcinome. Nos patients ayant consulté souvent tardivement, l’évolution a été fatale chez 2 patients (8,33%), 16 patients (66,67% étaient transférés en chirurgie pour des soins palliatifs et 5 patients (20,8%) sortis contre avis médical étaient perdus de vue.

## Conclusion

Le cancer de l’œsophage semble être en augmentation dans le service d'HGE du CHU Campus de Lomé. Ces caractéristiques épidémiologiques, diagnostiques et histologiques sont identiques à celles d'autres auteurs de par le monde. Son dépistage précoce passe par une consultation rapide devant toute dysphagie chez un sujet de 50 ans et plus, seul gage pour la réduction de sa fréquence.
